# Cure-All

**Published:** 1858-11

**Authors:** 


### CURE-ALL.

LORD GARDSTONE was an invalid, and made special inquiry after those persons who had testified to remarkable cures. He found that two-thirds of the number died very soon after they had given their certificates.

SIR ROBERT WALPOLE, LORD BOLINGBROKE, and WINNINGTON, were killed by persons who professed to accomplish wonders in the way of curing people of various diseases.

BISHOP BERKELEY believed that tar-water would cure half the ailments of men. He wrote a book in its praise, and confidently prescribed it for almost every ailment that came to his notice. One day he was taken sick himself. There was not an ounce of it in his house, and he died.

Let our readers remember, that only ignorant and presumptuous men talk of "certain" cures. The truly wise physician speaks with reserve, and such only ought to be trusted with human health and life.

